# Relation of neuropathologies to timely diagnosis of dementia in healthcare settings

**DOI:** 10.1002/alz70860_105791

**Published:** 2025-12-23

**Authors:** Yi Chen, Annie Chen, Melinda C Power, Francine Grodstein, Ana W. Capuano, Brittney S Lange‐Maia, Ali Moghtaderi, Emma K Stapp, Joya Bhattacharyya, Raj C. Shah, Lisa L. Barnes, David A. A. Bennett, Bryan D James

**Affiliations:** ^1^ Rush Alzheimer's Disease Center, Chicago, IL, USA; ^2^ The George Washington University, Washington, DC, USA; ^3^ Rush Alzheimer's Disease Center, Rush University Medical Center, Chicago, IL, USA; ^4^ The George Washington University, Washington DC, DC, USA

## Abstract

**Background:**

Multiple brain pathologies contribute to dementia. It remains unknown if certain neuropathologies are associated with susceptibility to diagnostic delays in dementia; delays are common and may result in missed opportunities for treatment and support. This study investigates relationships between presence of specific neuropathologies and timeliness of dementia diagnosis in healthcare settings.

**Method:**

Using five cohorts at Rush Alzheimer's Disease Center, we selected participants who had incident dementia based on annual cohort assessments, linkage to Medicare records, and later had postmortem brain autopsy. Neuropathologic examinations identified presence of Alzheimer's disease (AD), Limbic‐predominant age‐related TDP‐43 encephalopathy neuropathologic change (LATE‐NC), vascular, and Lewy bodies (LB) pathologies. In linked Medicare data, we defined timely dementia diagnoses as claims diagnoses received within 3 years prior to or 1 year after cohort dementia onset. We used logistic regression to assess associations of pathology with timely diagnosis versus underdiagnosis.

**Result:**

Among 548 participants (29% male, 95% non‐Latino White, mean[SD] age at dementia onset = 87.6[6.6] years, mean[SD] years from dementia onset to death = 3.8[3.2]), only 54% received a timely diagnosis in the healthcare settings. Adjusting for demographics, other pathologies, and time to death, we found AD (OR = 1.91, 95% CI = 1.21‐3.00) and LATE‐NC pathologies (OR = 1.83, 95% CI = 1.25‐2.68) were independently associated with higher odds of timely diagnosis. Vascular (OR=0.94, 95% CI = 0.55‐1.59) and LB pathologies (OR = 1.00, 95% CI = 0.64‐1.55) were not significantly associated with timely diagnosis. We observed no multiplicative interaction of coexisting AD and LATE‐NC pathologies (interaction term OR = 0.66, 95% CI = 0.26‐1.68).

**Conclusion:**

In deceased older adults with incident dementia, the healthcare system was twice as likely to capture those with AD and LATE‐NC pathologies in a timely manner. The reasons for this are unknown but may be related to differences in clinical manifestations. These findings provide some reassurance that those with AD pathology may be recognized in a timely window, which is believed to be necessary for effectively treating AD. The lack of association between vascular pathology and timely diagnosis may be concerning, given that treatments to improve vascular health (e.g. blood pressure control) can reduce cognitive impairment.

